# Assessing Geometry Perception of Direct Time-of-Flight Sensors for Robotic Safety

**DOI:** 10.3390/s25144385

**Published:** 2025-07-13

**Authors:** Jakob Gimpelj, Marko Munih

**Affiliations:** Laboratory of Robotics, Faculty of Electrical Engineering, University of Ljubljana, 1000 Ljubljana, Slovenia

**Keywords:** direct ToF, reflectivity, robotic safety, sensor evaluation metrics, sensor field of view, shape detection, single-point ToF, surface material effects, time-of-flight sensor

## Abstract

Time-of-flight sensors have emerged as a viable solution for real-time distance sensing in robotic safety applications due to their compact size, fast response, and contactless operation. This study addresses one of the key challenges with time-of-flight sensors, focusing on how they perceive and evaluate the environment, particularly in the presence of complex geometries and reflective surfaces. Using a Universal Robots UR5e arm in a controlled indoor workspace, two different sensors were tested across eight scenarios involving objects of varying shapes, sizes, materials, and reflectivity. Quantitative metrics including the root mean square error, mean absolute error, area difference, and others were used to evaluate measurement accuracy. Results show that the sensor’s field of view and operating principle significantly affect its spatial resolution and object boundary detection, with narrower fields of view providing more precise measurements and wider fields of view demonstrating greater resilience to specular reflections. These findings offer valuable insights into selecting appropriate ToF sensors for integration into robotic safety systems, particularly in environments with reflective surfaces and complex geometries.

## 1. Introduction

Time-of-flight sensors have been increasingly used in such various fields as consumer electronics, industrial automation, and autonomous systems thanks to their compact, cost-effective, and efficient method of depth measurement. Their small size and low power consumption make them a practical choice for modern devices that require fast and accurate spatial data. They are becoming especially useful in robotics, where rapid and precise depth sensing is very critical for safe and efficient operation. With robots increasingly entering our daily lives and operating in shared environments alongside humans, ToF sensors may enhance safety and promote further human–robot collaboration.

ToF sensors estimate distance from signal round-trip time, and are commonly grouped into indirect ToF (iToF) and direct ToF (dToF) variants [1]. iToF sensors infer depth from the phase shift of a modulated optical carrier, whereas dToF sensors measure the arrival time of a nanosecond pulse. Commercial iToF modules offer low <10 mW power usage while delivering centimeter-level accuracy at ranges up to 3 m @ 30 fps, whereas recent SPAD-based dToF sensors extend precision beyond 5 m and suppress multipath artifacts [2,3]. Both types are widely used in robotics for navigation and object detection as well as increasingly in safety-critical tasks such as collision avoidance.

As collaborative robots leave fenced cells [4] and robots and humans begin to share workspaces, safety sensing has progressed from fixed guards to volumetric depth devices such as safety-rated 2D LiDAR scanners and stereo/iToF cameras for workspace monitoring [5]. While these devices deliver rich point clouds, they do so at an order-of-magnitude higher cost and compute load than single-point ToF modules [6]. These sensors provide discrete one-dimensional distance measurements from a compact module, allowing for high sampling rates. When several of these 1D sensors are arranged in a ring or strip directly on robotic arms, it enables the acquisition of real-time proximity data crucial for implementing rapid responses and adaptive speed control to prevent collisions [7]. The protective capabilities of such ToF-based systems can be effectively augmented by complementary sensor modalities; for instance, capacitive sensors offer enhanced near-field detection capabilities [8], while inductive sensors can provide definitive contact confirmation [9]. A commercial example is SICK’s End-of-Arm-Safeguard, a ring-shaped ToF array designed for robot flange mounting. With its specified 14° field of view and 110 ms response time, this system establishes a dynamic protective field that preemptively halts robot motion before physical contact, significantly bolstering safety in collaborative environments [10].

Performance degrades under high ambient illumination (more than 20 kilolux ), particularly sunlight, as the infrared content can saturate the sensor’s photo detector array. This effectively raises the noise floor, making it difficult to distinguish the sensor’s returning light pulse. As a result, the signal-to-noise ratio is lowered, which can shorten the usable range or lead to inaccurate measurements. Performance is also affected by surface textures and in self-occluded zones created by the arm geometry [11]. While some approaches in the literature address such issues for standard cameras by analyzing color and texture distortions [12], these methods are not applicable to ToF sensors, which do not capture this type of information.

Self-occlusion, when the robot itself blocks the sensor’s view, can cause blind spots; this can be addressed with simulations to optimize sensor placement [13] or by using robot kinematics to detect and discount self-occluded areas via precomputed reference distances [14].

In addition to occlusion, surface properties have a major effect on accurate data acquisition. Highly specular targets can redirect the emitted beam away from the receiver, producing depth voids or gross over-range errors. To counter this, combining shape-from-polarization techniques with ToF depth data have been proposed [15]. Other studies have applied mathematical correction models [16], AI-driven simulations for error correction, or signal processing approaches such as adaptive filtering [17] and transient data transformations [18]. Combining multiple ToF sensors with varied integration times has also been shown to reduce errors across surfaces of differing reflectivity [19] by using sensor fusion to mitigate signal noise and improve accuracy.

In order to deploy ToF sensors in robust safety systems, it is essential to understand how surface geometry and material properties affect their performance. For this purpose, the sensors’ perception abilities need to be tested and evaluated under conditions that closely replicate their intended operational environments by using common object shapes and materials that they are likely to encounter. Despite their widespread use in robotics, there is a lack of studies examining how ToF sensors perceive and interpret different object geometries, shapes, and sizes in conjunction with material properties within robotic workspaces. Most of the literature concentrates on how reflectivity alone affects range error; in addition, shape-related work is almost always carried out with full 3D ToF cameras rather than with simple point-range units.

In this paper, we present a detailed study of how single-point time-of-flight sensors perceive a robot workspace that contains objects of different sizes, shapes, and materials. By evaluating the interaction between ToF sensor measurements and scene geometry, including fundamental shapes such as cylinders which serve as a simplified approximation for human limbs, we highlight key detection behaviors and limitations. This work provides practical insight into time-of-flight sensor perception and offers guidance for improving safety in collaborative systems where robots and humans share a workspace.

## 2. Methodology

To accurately evaluate and compare how the ToF sensors perceive the shape of the environment, a reproducible test setup was designed to let each sensor measure the same scenes under controlled conditions. This setup enables systematic data collection, ensuring that the results reliably reflect each sensor’s characteristics. In this section, we describe the sensors used along with the measurement setup, data acquisition procedures, data evaluation methods, and metrics employed to quantify sensor performance.

### 2.1. Sensors Description

Among the broad range of available ToF sensors, we selected two from different manufacturers, with distinct operating principles and technical characteristics.

The VL53L4CD from STMicroelectronics, Geneva, Switzerland, is a single-pixel direct time-of-flight sensor. It uses a Vertical-Cavity Surface-Emitting Laser (VCSEL) emitter at a wavelength of 940 nm and a Single-Photon Avalanche Diode (SPAD) array detector. It bins the returns from the SPAD array into a single range measurement per emission cycle, enabling accurate distance readings from 1 mm to 1300 mm. The sensor provides a diagonal field of view of 18° and operates at measurement frequencies up to 100 Hz [20].

The AFBR-S50MV85I from Broadcom Inc., Steinhausen, Switzerland, is a multi-pixel ToF sensor module designed for rapid and accurate distance measurements. It uses a VCSEL emitter operating at a wavelength of 850 nm and a 32-pixel SPAD-based receiver array arranged in an 8 × 4 hexagonal grid, giving it a field of view of 12.4° × 5.4°. This sensor has a measurement range of up to 10 m, which varies according to target reflectivity and ambient conditions. It can reach measurement frequencies up to 3 kHz in a single-point measurement configuration [21].

**Figure 1:**
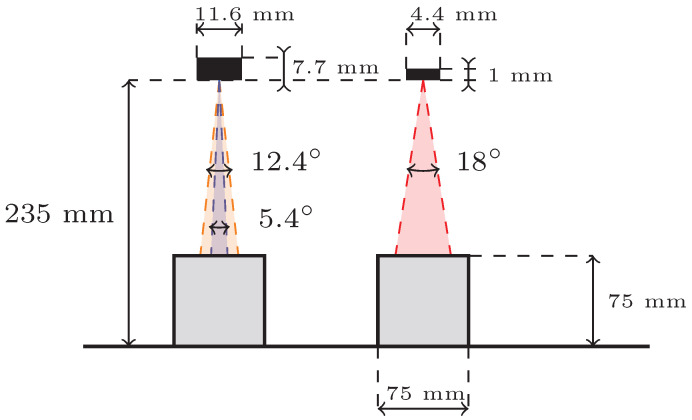
Field of view representation of the different sensor configurations. The left side shows the 12.4° and 5.4° FoV sensor configurations, while the right side shows the 18° FoV configuration.

The two sensors were chosen for their comparable fields of view to allow for relevant comparisons, as illustrated in Figure 1. Because one sensor features a rectangular field of view, the experiments were conducted using three distinct setups, which will henceforth be referred to as the 18° FoV, 5.4° FoV, and 12.4° FoV sensor configurations.

### 2.2. Sensor Mounting and Configuration

**Figure 2:**
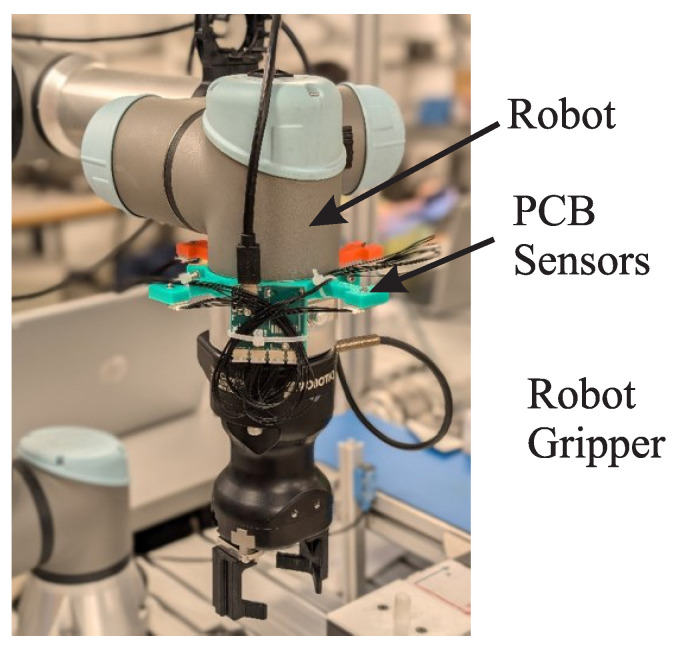
A ring of VL53L4 ToF sensors mounted on a UR5e robot.

All sensors were mounted on a robot arm. The 18° FoV sensor units were installed using a custom circular ring mount capable of holding up to four sensors, positioned flush with the mount surface and facing forward along the robot arm segment. To minimize self-occlusion, the sensor mounts were extended outward from the arm’s central axis, as shown in Figure 2. They connected via I^2^C to a PCB with an ARM Cortex M7 microcontroller which managed sensor operations. The PCB communicated with a PC over a USB serial interface. Preliminary tests showed no interference between sensors even with overlapping fields of view. Nonetheless, measurements were triggered sequentially with an 11 ms cycle to eliminate any potential interference. Results were aggregated into a single message per cycle, which was transmitted to the PC at a final sampling rate of 90 Hz.

The 12.4° and 5.4° FoV sensor configurations were mounted on a custom shield for an STM Nucleo board, also connected to the PC via USB. A single sensor could reach approximately 2 kHz when transmitting only depth data. To match the 18° FoV sensor configuration, four sensors were integrated on the shield and sent individual depth readings along with unique IDs and timestamps to the PC. Due to bandwidth limitations, the combined sampling rate was reduced to 190 Hz. Under the laboratory lighting and timing conditions used in this study, we did not detect any noticeable optical crosstalk between the four sensors.

### 2.3. Robot and Workspace Configuration

Each sensor configuration was mounted individually, one at a time, onto a Universal Robots UR5e collaborative robotic arm which served as the central component of the experimental setup. Measurements were conducted sequentially, first with one sensor configuration and then repeated with the other. Due to slightly different mounting positions affecting the sensor heights, the robot’s linear trajectory was adjusted accordingly for each configuration to ensure identical sensor-to-ground distances. Additionally, X-axis offsets were accounted for during subsequent data alignment.

### 2.4. Calibration

Sensor calibration was performed after the units were fixed to the robot flange. The robot moved to a hard-coded pose in which the sensors faced a white matte at 310 mm ± 0.1 mm. That reference distance was entered into each sensor’s vendor API, which internally adjusts the timing offset. All calibration and subsequent measurements were taken in an indoor lab under constant lighting conditions; no daylight was present, so the ambient illumination remained unchanged for the duration of the experiments.

### 2.5. Test Objects

To evaluate the way the ToF sensors see in realistic environments, objects with varying shapes, sizes, and materials were placed in the work area and measured. Three primary geometries were selected: cuboid, ramp, and cylinder, as presented in Table 1. The ramp and cylinder objects were made of 3D-printed white plastic, while the cuboids varied in material and included 3D-printed plastic, aluminum, unpolished wood, and polished wood with a reflective coating. The aluminum and polished wooden surfaces introduced specular reflection conditions to assess sensor responses under reflective scenarios. These materials were specifically chosen to represent a sample of materials commonly found in industrial settings where robotic arms typically operate alongside humans.

The dimensions of the test objects were also varied to assess sensor detection capabilities across different object sizes, an essential aspect of evaluating their effectiveness in safety applications. Multiple cylinders with varying diameters were included, and the cuboid dimensions were altered by stacking several units both horizontally and vertically. This approach enabled evaluation of sensor performance on objects commonly found in the robot environment, whether tall and narrow or short and wide.

All test objects were placed flat on the workspace surface and oriented so that their longest dimension (length) was perpendicular to the robot’s direction of travel. In this way, measurements focused exclusively on the width and height of the objects within the sensors’ field of view. All objects had lengths exceeding 150 mm, ensuring that the objects fully extended beyond the sensors’ field of view, thereby eliminating any influence of object length on the sensor data.

### 2.6. Measurement Combinations

This section describes the different object arrangements used in the experiments. The first scenario evaluated how the sensors perceive increasing object width. Cuboids measuring 45 mm in width were placed one by one side-by-side without gaps. A separate measurement pass was conducted for each configuration, starting with a single cuboid and adding one more for each subsequent run, up to a total of five cuboids (225 mm total width).

The second scenario focused on vertical stacking. Here, 45 mm wide cuboids were stacked one by one on top of each other. Similar to the first scenario, a separate measurement with the robot passing over the target area and objects was performed for each stack height, starting with one cuboid on a flat surface and increasing to a maximum of four, reaching a maximum height of 180 mm.

In the third scenario, the sensors were tested with cylindrical objects of varying diameters (ranging from 25 mm to 100 mm) placed in the same location. This setup combined variations in both object width and height to assess the sensors’ response.

In the sixth scenario, the impact of background color on sensor performance was tested by repeating the shape differentiation setup (scenario five) with the workspace background surface changed to black.

Finally, the seventh scenario considered different material types: 3D-printed plastic, unpolished wood, polished wood with a reflective coating, and aluminum. All objects were cuboids with a width and depth of 75 mm. This allowed for evaluating sensor accuracy under varying reflectivity and surface textures.

### 2.7. Data Collection

Data samples from both sensor setups were recorded directly onto a PC using a Python 3.8.10 script and saved in CSV format. Each sample included the measured distance, a sensor identifier, and a timestamp.

**Figure 3:**
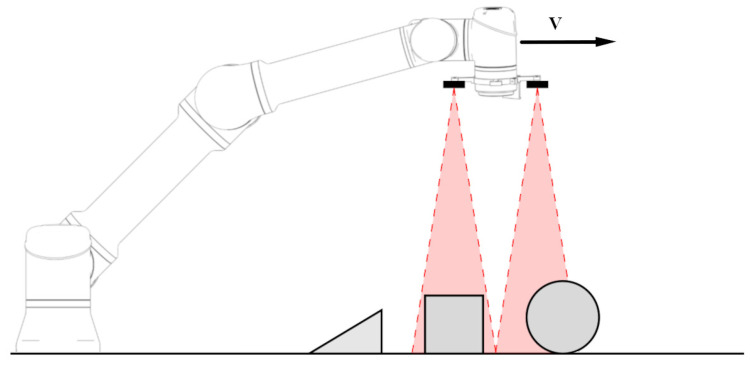
Diagram illustrating the data collection process, where the robot arm moves the sensors at a constant velocity (V) over a series of test objects.

The robotic arm followed linear trajectories at predefined constant velocities across the workspace containing the test objects, as illustrated in Figure 3. During each measurement pass, the robot arm with the sensors moved over the test objects, which were placed on a flat surface. All scenarios were first conducted using the sensor configuration with the symmetrical 18° field of view (FoV), then repeated with the sensor configuration providing the elliptical 12.4° × 5.4° FoV. Due to its asymmetric field of view, the latter was tested in two orientations for each scenario: first in its default orientation (5.4° effective FoV on horizontal scans) and again after being rotated by 90° (12.4° effective FoV on horizontal scans). This procedure enabled a comparative evaluation of how the field of view’s shape and orientation influence the sensor’s detection performance.

### 2.8. Synchronization

Synchronization of samples from either sensor was essential for accurate comparative analysis. Initially, synchronization was performed using a robot-generated trigger signal sent when the robot reached a constant predefined velocity. Some inconsistencies due to timing jitters were observed; thus, the first samples of successive runs were captured a few milliseconds apart, meaning that consecutive passes did not align perfectly. To address this, a thin plastic sheet covering the sensors’ fields of view was introduced at the starting position at the beginning of each measurement, serving as a physical landmark. As the robot moved forward, the sensors moved past this sheet, causing a distinct and sharp change in sensor readings. This consistent landmark signal enabled precise alignment of sensor data and ground truth distances across multiple measurements.

### 2.9. Data Processing

The acquired sensor data were grouped by scenario and aligned using the previously described synchronization methods. Both recordings were re-sampled to a common frequency of 200 samples per second using linear interpolation in order to standardize the data length and sampling rates. This step ensured that the initial difference in hardware sampling rates (90 Hz vs. 190 Hz) did not introduce bias into the final comparative analysis, as all profiles were evaluated with an identical spatial resolution. For clarity, sensor readings were plotted relative to the robot’s traveled distance. Synthetic ground truth signals were generated for each measurement based on measured sensor–object distances, object dimensions, spacing, and distance from the reference landmark.

Prior to comparative analysis, postprocessing normalization was applied to account for differences in object size and recording length. Because measurement error was defined as the difference between sensor readings and ground truth, extended sequences of ground-level data introduced bias. Although such readings had consistent low noise, they outweighed the object-related data and risked distorting the results. To avoid this, each sequence was cropped to focus on the object region, beginning 50 mm before the leading edge of the first object and ending 50 mm after the trailing edge of the last. Inspection of all recordings showed that the sensor signal stabilised within roughly 35–40 mm on either side of each object; therefore, a 50 mm margin was used to guarantee that the entire transition zone was included without retaining excessive ground-level data.

### 2.10. Performance Metrics

Object measurements across all scenarios were quantitatively evaluated using sensor data and corresponding ground truth values at each data point, applying several performance metrics. These metrics capture different aspects of sensor behavior and are commonly used in sensor performance evaluation [22].

The Mean Squared Error (MSE), a metric that inherently emphasizes larger errors, was used to assess the average squared deviation between the sensor data and ground truth values. Its square root, the Root Mean Square Error (RMSE), was also calculated to express the typical magnitude of the error in the original measurement units, providing an overall sense of how closely the measured signal follows the ground truth. The Mean Absolute Error (MAE) provided the average absolute deviation, offering a general indication of signal alignment that is less influenced by outliers compared to MSE. The Standard Deviation (STD) of the error, computed around the mean error, was used to represent the variability of the sensor readings, indicating their consistency [23].

The Absolute Sum of Errors (ASE) captures the total magnitude of all deviations regardless of direction. Finally, the Area Difference (AD) reflects the net bias by indicating whether the sensor tends to overestimate or underestimate the measured values.

To account for varying measurement lengths, the RMSE, MSE, and MAE were normalized by the total measurement interval. The ASE and AD were normalized with respect to the total area under the ground truth curve.

The mathematical formulations for these metrics are as follows:

(1) $$\begin{array}{cc} \mathrm{MSE} & =\frac{1}{N}\sum_{i=1}^{N}{\left(y_{i}-{\hat{y}}_{i}\right)}^{2} \end{array}$$

(2) $$\begin{array}{cc} \mathrm{RMSE} & =\sqrt{\frac{1}{N}\sum_{i=1}^{N}{\left(y_{i}-{\hat{y}}_{i}\right)}^{2}} \end{array}$$

(3) $$\begin{array}{cc} \mathrm{MAE} & =\frac{1}{N}\sum_{i=1}^{N}\left|y_{i}-{\hat{y}}_{i}\right| \end{array}$$

(4) $$\begin{array}{cc} \sigma_{e} & =\sqrt{\frac{1}{N}\sum_{i=1}^{N}{\left(e_{i}-\bar{e}\right)}^{2}} \end{array}$$

(5) $$\begin{array}{cc} \mathrm{ASE} & =\sum_{i=1}^{N}\left|y_{i}-{\hat{y}}_{i}\right| \end{array}$$

(6) $$\begin{array}{cc} \mathrm{AD} & =\int_{a}^{b}\left(y\left(t\right)-\hat{y}\left(t\right)\right)\;dt. \end{array}$$

where for Equations (1)–(5):

- *N* is the total number of data points;
- $y_{i}$ is the ground truth value at data point *i*;
- ${\hat{y}}_{i}$ is the sensor’s measured value at data point *i*;
- $e_{i}=y_{i}-{\hat{y}}_{i}$ is the error at data point *i*;
- $\bar{e}=\frac{1}{N}\sum_{i=1}^{N}e_{i}$ is the mean error;

and for Equation (6):

- y(t) is the ground truth signal as a function of *t*;
- $\hat{y}\left(t\right)$ is the sensor’s measured signal as a function of *t*;
- The integral is taken over the measurement interval from *a* to *b*.

## 3. Results

This section presents the experimental results from a series of controlled scenarios designed to assess how each of the two dToF sensors perceives object shapes, dimensions, and surface materials in the context of robotic safety. The measurements cover horizontal and vertical size variation using stacked cuboids, changes in object geometry using cylinders of increasing diameter, and spatial separation through varying gap widths between identical objects. Additional tests include the perception of different geometric shapes at varying speeds and background contrasts as well as the influence of surface material reflectivity on distance measurements. A broad set of measurements was collected during testing; of these, only a selection of typical cases is presented in the following figures to better illustrate the results and maintain clarity.

Each figure shows sensor measurements from the three primary field of view configurations we evaluated, namely, the 5.4° FoV, 12.4° FoV, and 18° FoV configurations, plotted against the robot’s traveled distance (x-axis, mm) and sensor readings (y-axis, mm). To improve readability and provide a more intuitive interpretation of the distance measurements, the y-axis is inverted; this places the origin effectively at the top left, meaning that greater sensor-measured distances are plotted downwards, with the ground level (representing the furthest detected distance) forming the bottom baseline of the graph and objects appearing as topographical elevations from this baseline. A black dashed line represents the actual object shape, serving as the synthetic ground truth.

**Figure 4:**
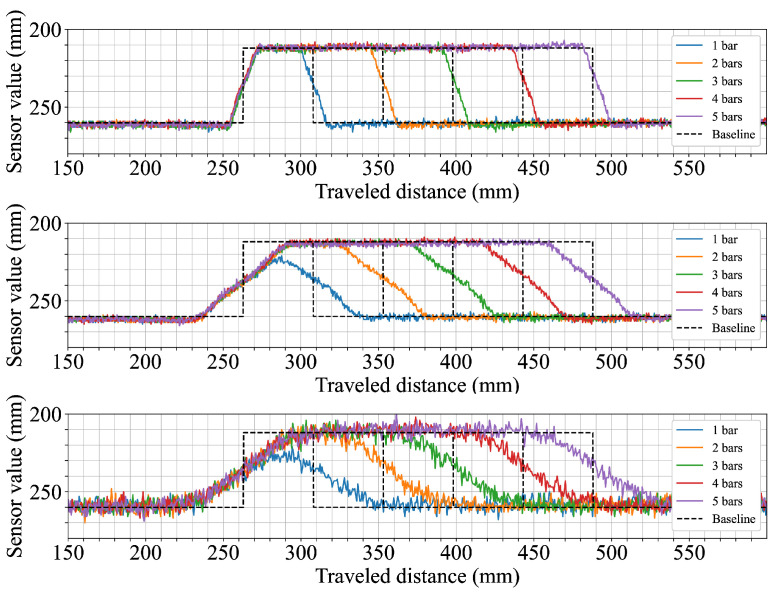
Horizontally stacked 45 × 45 mm cuboids. Each subplot shows results from a different field of view configuration: the top row corresponds to the 5.4° FoV sensor configuration, the middle row to the 12.4° FoV, and the bottom row to the 18° FoV.

The first scenario explored how the sensors respond to objects of increasing width. This setup aimed to evaluate how well each configuration captures edge transitions and shape fidelity as the object’s horizontal dimension expands. As shown in the first row of Figure 4, the sensor configuration with the narrow 5.4° FoV provided the clearest profile, closely following the ground truth and exhibiting sharp transitions at object edges. In contrast, both 12.4° FoV and 18° FoV configurations underestimated the object height when measuring a single cuboid by around 5 mm and 10 mm, respectively. From two cuboids onward, their measurements more closely approximated the expected levels, although the transition zones remained broader.

**Figure 5:**
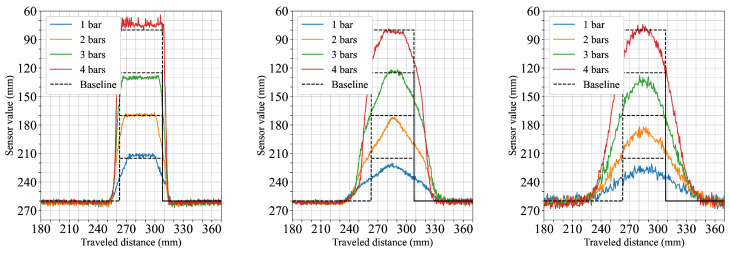
Vertically stacked 45 × 45 mm cuboids. From left to right, the subplots correspond to the 5.4° FoV, 12.4° FoV, and 18° FoV configurations, respectively.

The second scenario focused on vertical stacking to observe how the sensors respond to changes in object height while maintaining constant width. Figure 5 presents the distance profiles for each configuration as the stack height increases from one to four cuboids. The narrow 5.4° FoV sensor configuration captures distinct plateaus at each height level with well-defined transitions. In contrast, the wider 12.4° and 18° FoV configurations show increasing alignment with the ground truth as the stack becomes taller; however, the object edges appear more rounded and the transitions are broader compared to the narrower FoV configuration.

**Figure 6:**
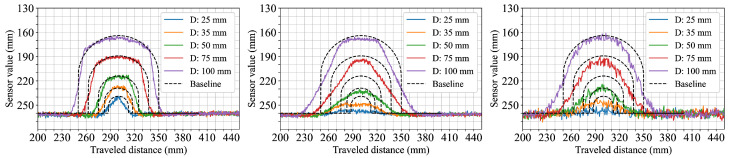
Plastic cylinders with diameters ranging from 25 mm to 100 mm. From left to right, the subplots correspond to the 5.4° FoV, 12.4° FoV, and 18° FoV sensor configurations, respectively.

Cylindrical objects of varying diameter were used in the third scenario to observe how each sensor configuration captures curved shapes with simultaneous changes in width and height. Figure 6 shows the resulting measurements for cylinders ranging from 25 mm to 100 mm in diameter. The 5.4° FoV configuration records distinct object boundaries and follows the curvature of each cylinder, with peak measured heights closely matching the ground truth. The 12.4° FoV configuration produces wider transitions and lower peak heights, particularly for the smaller diameters. The 18° FoV configuration shows increased profile smoothing and shape variation; the smallest cylinder (D: 25 mm) is only barely captured, appearing as a small bump with substantial deviation from the expected contour.

**Figure 7:**
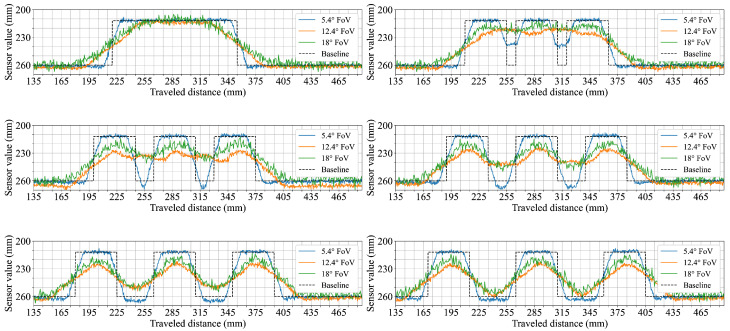
Horizontally stacked 45 × 45 mm cuboids with varying separation distances. The six subplots show measurements for different gap widths: 0 mm (**top left**), 10 mm (**top right**), 20 mm (**middle left**), 30 mm (**middle right**), 40 mm (**bottom left**), and 50 mm (**bottom right**). In each subplot, the colored lines correspond to the different sensor configurations.

The fifth scenario focused on how increasing separation between adjacent objects is represented in the distance measurements. The goal was to observe how each sensor configuration captures gaps of different widths and at what point individual objects become distinguishable. Figure 7 shows cases of the recordings for gap widths ranging from 0 mm to 50 mm. With no gap (0 mm), all configurations produce continuous profiles similar to earlier measurements of horizontally stacked cuboids. At a 10 mm gap, the 5.4° FoV configuration shows medium dips in the profile between cuboids, while the wider 12.4° and 18° FoV sensor configurations exhibit only slight reductions of less than 5 mm in top-level readings. At a gap of 20 mm, a more distinct depression reaching to ground level appears in the data from the 5.4° FoV configuration, while the wider FoV configurations begin to show slightly deeper dips. For gaps of 30 mm and 40 mm, the separation between cuboids becomes more prominent in all configurations, though the two wider FoV sensors never reach the ground level. At 50 mm, all three configurations show complete dips to ground level between the cuboids, with individual objects fully distinguishable.

**Figure 8:**
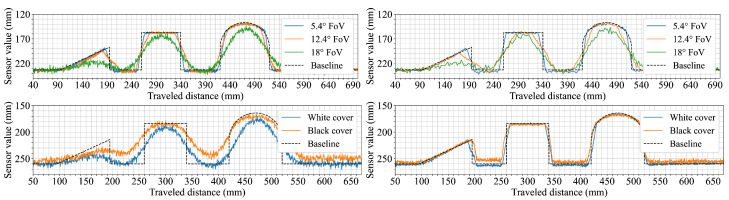
Sensor responses to three sequentially placed objects (a 100 mm wide, 45 mm tall plastic ramp, a 75 × 75 mm plastic cuboid, and a 100 mm diameter plastic cylinder) under different conditions. The top row shows measurements taken at two different travel speeds (60 mm/s and 250 mm/s) with each colored line representing a different sensor configuration, while the bottom row shows measurements taken at 60 mm/s over two different ground background colors (white and black).

Scenario six was designed to observe how movement speed affects shape representation in the distance profiles. The arrangement of multiple geometric shapes allows for direct comparison between slow and fast traversal speeds. The top row of Figure 8 shows the results for both speeds. At 60 mm/s, the 5.4° FoV configuration captures distinct profiles for all three shapes, including clearly defined edges. The 12.4° configuration reflects the same sequence but with visibly smoother transitions. The 18° configuration shows flatter profiles with reduced contrast between shapes and limited visibility of the ramp. At 250 mm/s, the general shape and order remain consistent for each configuration but the transitions broaden slightly, particularly in the data from the 12.4° FoV configuration.

In a variation of the previous scenario, the background surface was changed to evaluate the effect of background color on measured distances. The shapes remained identical, allowing for a direct comparison of background influence. The bottom row of Figure 8 shows that ground-level readings across all configurations are shorter on the black surface. The reduction is approximately 5 mm for the 5.4° and 12.4° FoV configurations, while the 18° FoV sensor configuration shows a larger shift exceeding 10 mm. Despite this offset, the profiles of the individual shapes remain similar in form, with the 18° FoV configuration recording object heights that are closer to the expected values than in the white background condition.

**Figure 9:**
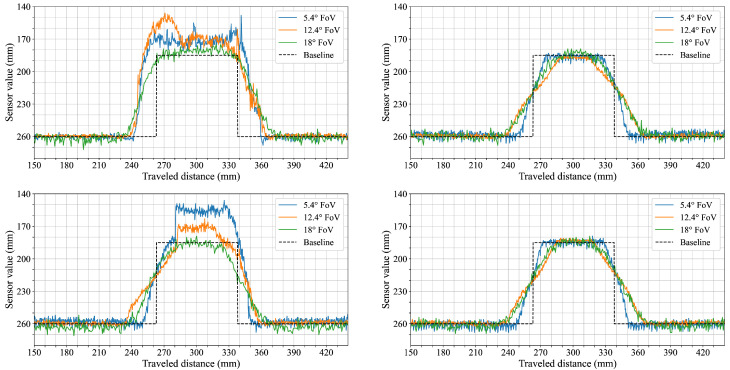
Cuboids with the dimension of 75 × 75 mm made of four different materials: aluminum (**top left**), plastic (**top right**), coated wood (**bottom left**), and uncoated wood (**bottom right**). Each colored line corresponds to a different sensor configuration.

The seventh and final scenario aimed to observe how changes in surface reflectivity and texture affect the acquired data by using objects of identical shape and size made from different materials. Figure 9 shows the measured profiles for aluminum, uncoated wood, reflective-coated wood, and 3D-printed plastic across the three sensor configurations. The uncoated wood and plastic objects produce consistent results across all sensors, with profiles similar to those observed in earlier tests on diffuse surfaces. For the aluminum object, both the 5.4° and 12.4° FoV configurations display increased variation in the signal, with the 12.4° configuration showing a noticeable hump in the profile. The 18° FoV configuration remains comparatively stable, with broader transitions but no significant distortion. With the reflective-coated wood, the the 5.4° and 12.4° FoV configurations show abrupt jumps and irregularities in the measured profile, while the 18° FoV configuration again produces a smoother output that is comparable to the results from the uncoated wood except for slightly wider transitions.

## 4. Discussion

This section interprets the experimental results, emphasizing how dToF sensors perceive object geometry under various conditions, including differences in shape, size, surface material, and reflectivity. Key trends and anomalies are discussed in detail, supported by visual and quantitative analysis. The goal is to highlight the practical implications of these findings for informing the deployment strategies of dToF sensors in robotic safety applications based on their perceptual characteristics.

From the results, it is evident that the sensor FoV significantly impacts shape acquisition accuracy. Narrow FoVs, as exemplified by the 5.4° FoV configuration, consistently deliver sharper transitions and more accurate shape profiles across various scenarios. In contrast, wider FoVs such as those represented by the 12.4° and 18° FoV configurations exhibit broadened transitions and reduced spacial accuracy due to averaging effects. This trend was evident in the first two scenarios (Figure 4 and Figure 5), where sensor configurations utilizing wider FoVs initially reported lower object heights. This underestimation was reduced when more objects were stacked horizontally, as the larger visible surface area helped to counteract the averaging effect. The perceptual limitations imposed by wider FoVs were further confirmed in the cylinder scenario (Figure 6). Here, the widest FoV configuration tested (18°) failed to detect the smallest cylinders (25 mm), likely because the object’s dimensions were small relative to the sensor’s large instantaneous field of view, causing the return signal from the object to be overwhelmed or excessively diluted by signals from the background. Additionally, in the fifth scenario (Figure 8) measurements from the 18° FoV configuration showed that the perceived profiles of the cuboid and cylinder became very similar, demonstrating how wider FoVs tend to round off distinct geometric features.

This phenomenon of spatial averaging in dToF sensors not only degrades overall shape fidelity but also critically limits their ability to detect fine environmental features such as gaps between objects. In the gap detection scenario (Figure 7), the sensor configuration with the narrowest FoV (5.4°) demonstrated the capability to clearly identify gaps as narrow as 10 mm, fully resolving them (i.e., measuring down to the ground plane) at 20 mm. In comparison, the configuration employing the widest FoV (18°) required the gap to widen to approximately 50 mm before its signal registered the ground surface between the objects. This illustrates how a larger FoV can effectively ’bridge’ or fail to discern smaller empty spaces due to averaging over a wider area. Such limitations become particularly critical in safety-sensitive robotic applications when the specific safety concept mandates the reliable detection of small obstacles or narrow passages, as their non-detection could lead to hazardous operational failures. Similar challenges have been reported in other ToF sensor studies, with wide-angle configurations being shown to reduce the ability to resolve fine object features and edge details in structured environments [24].

The sensor’s perception of object geometry appeared to be only mildly influenced by the tested movement speeds. A slight broadening of transitions at higher speeds was observed in the measurements from the 12.4° FoV configuration (Figure 8). Changing the background surface contrast (from white to black) had a more noticeable impact on the perceived distances (Figure 8). Specifically, the measured distance to the ground was consistently shorter for all tested configurations when the background was black. This suggests that surface reflectivity, even of the background, can influence the baseline distance readings of sensors. This effect of a shorter perceived ground distance was most pronounced for the 18° FoV configuration. Interestingly, this shift in the perceived ground plane also resulted in its object height readings (relative to this new baseline) appearing closer to the actual object heights. In contrast, the configurations with FoVs of 5.4° and 12.4° exhibited measurements that remained comparatively stable despite changes in background color, indicating potentially different internal signal processing or optical characteristics influencing their response to overall scene reflectivity changes under these conditions.

Surface material properties, particularly high reflectivity, were found to introduce notable distortions in the perceived geometry. This was especially evident in the measurements from the 5.4° and 12.4° FoV configurations when encountering specular reflective surfaces (Figure 9). In particular, the 12.4° FoV sensor configuration exhibited a particularly clear and consistent undershot in its measured profile when scanning these reflective objects. This consistently appeared on the entry side of the object, and depended on the direction in which the sensor moved across the surface. This undershot artifact was caused by the high-amplitude return signal overwhelming the sensor’s electronics. The high-level reflection can saturate the sensor’s linear dynamic range even after its Automatic Gain Control (AGC) has stabilized at a minimum setting. This persistent over-driving of the receiver distorts the return pulse shape, which is then misinterpreted as a shorter time of flight, resulting in an erroneously short distance reading. These effects were not observed with diffuse matte surfaces such as uncoated wood or plastic. Similar distortion artifacts caused by reflectivity have been observed in prior work using ToF sensors and cameras [25], where specular surfaces led to localized errors and depth inconsistencies.

Interestingly, the sensor configuration with the widest FoV (18°) yielded more stable profiles when measuring the same reflective materials. Although its perceived transitions remained wider than those recorded for matte surfaces (a characteristic consistent with its wider FoV), this particular sensor configuration did not exhibit the same pronounced localized shape distortions. This greater resilience is directly linked to the saturation phenomenon. Its wider FoV spreads the emitted light over a larger area, resulting in a lower power density. This produces a less intense return signal detection that is less likely to saturate the electronics, effectively diluting the impact of the localized specular returns that dominated the readings of the narrower FoV sensors.

**Figure 10:**
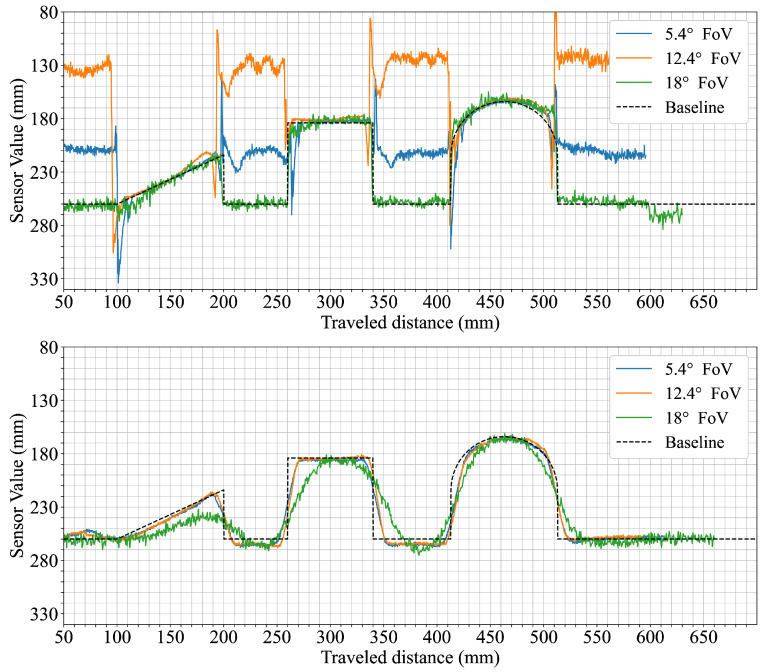
Sensor response to extreme surface conditions. The top subplot shows the measurement of three plastic objects (a plastic ramp with 100 mm width and 45 mm height, a 75 × 75 mm plastic cuboid, and a 100 mm diameter plastic cylinder) placed on a highly reflective mirror surface. The bottom subplot shows the results for the same objects placed on a highly dissipative diffuse black fabric surface.

To further investigate the impact of extreme surface properties, additional experiments were conducted using a highly reflective mirror and a diffuse black fabric as the surface on which the three geometric shapes were placed. The results are presented in Figure 10.

Because it is an extreme specular reflector, the mirror surface amplified the previously noted issues for the two narrower FoV configurations. Readings from the mirror background showed strong deviations which diminished the sensors’ ability to reliably detect the objects. The transitions between the objects and the mirror also exhibited sharp spikes, representing both under- and over-estimations of distance as the AGC struggled to keep up with the fast reflectivity changes. In stark contrast, the performance of the 18° FoV configuration was very stable and even improved its accuracy in object perception. On the other hand, the diffuse black fabric showed results consistent with the black background test from scenario six (Figure 8).

**Figure 11:**
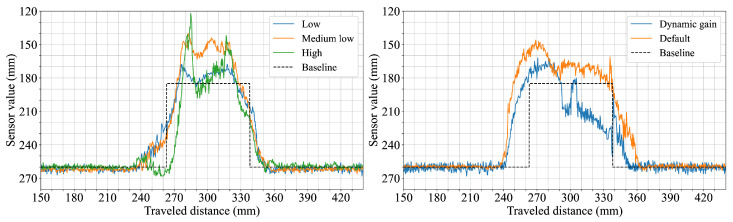
A 75 × 75 mm aluminum block measured with the 12.4° FoV sensor configuration with different gain settings. Each color graph represents a different gain configuration.

To explore potential mitigation strategies for such reflectivity-induced perceptual distortions in sensors, adjustments to the internal sensor gain control settings were investigated using the 12.4° FoV sensor configuration. The influence of these changes is shown in Figure 11. Higher static gain settings amplified measurement fluctuations, generating spikes, while lower gain settings reduced fluctuations but compromised object height accuracy. Given the safety-critical context, settings such as default gain that may lead to an underestimation of true object distances (i.e., reporting objects as being closer than they are) could be considered preferable in this instance. Such behavior would bias the system towards safer ′false positive’-type protective actions (e.g., a robot stopping earlier than strictly necessary) rather than risking dangerous ‘false negative’ scenarios (e.g., failing to detect an obstacle or detecting it too late).

In contrast to our dToF results, indirect Time-of-Flight (iToF) sensors infer distance from the phase shift of a return signal. This reliance on phase makes them inherently more susceptible to errors from multipath interference occurring when reflections from multiple surfaces mix at the sensor as well as from specific surface properties of the target such as material and color [26]. Therefore, the distortions we observed on specular objects (Figure 9) would likely be far more pronounced with iToF sensors.

**Figure 12:**
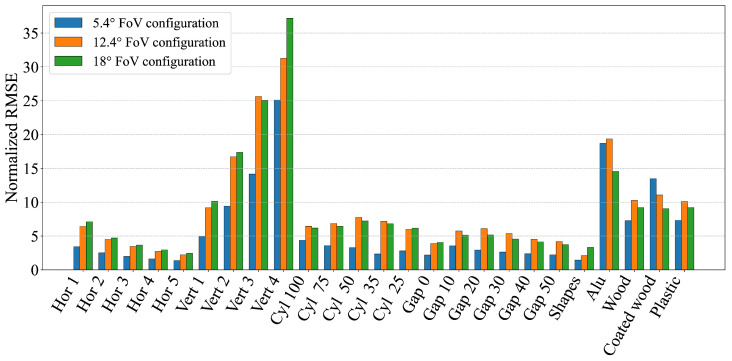
Graph showing the RMSE of all measurements normalized by the length of the measurement interval.

**Figure 13:**
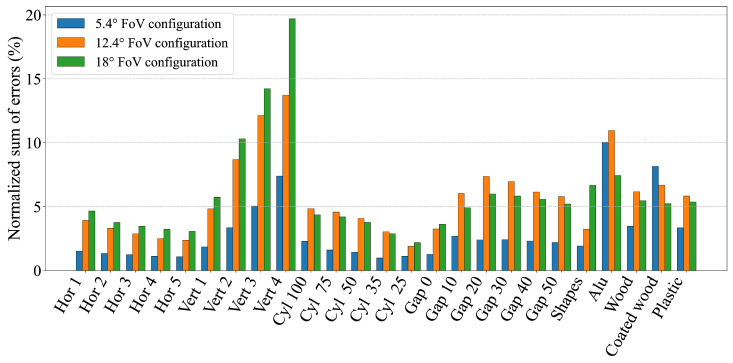
Graph showing the absolute sum of errors for all measurements normalized by the total area under the ground truth.

To further understand how different environmental conditions and object characteristics affect the geometric perception accuracy of dToF sensors, the measurements from each scenario were quantitatively evaluated. This was achieved by assessing each scenario against a set of performance metrics (RMSE, MAE, MSE, standard deviation, and area-based errors) in both absolute and normalized forms. The scenarios were then ranked for each metric and these individual rankings were averaged to produce a single overall performance score. Table 2 summarizes these evaluations by listing the five best- and worst-performing scenarios for each sensor configuration based on this averaged ranking. The results of this analysis indicate that, in general, dToF sensors achieve more accurate geometric perception in scenarios involving relatively large and continuous surfaces, such as horizontally stacked multiple cuboids (e.g., ′Hor 5,’ ′Hor 4’). Conversely, scenarios presenting significant perceptual challenges included those involving sharp and substantial height changes (e.g., ′Vert 3’, with vertically stacked cuboids leading to narrow observable top surfaces from a distance) or those with strong specular reflections (e.g., aluminum and coated wood objects). These scenarios consistently resulted in less accurate geometric perception across all tested configurations, requiring sensors to measure sharp and significant height changes or to manage strong specular reflections, thereby amplifying measurement errors despite similar transition zones. This general trend is further illustrated by the aggregated error metrics in Figure 12 (normalized RMSE) and Figure 13 (normalized ASE). These figures show how scenarios involving, for example, highly reflective materials or geometries with small dimensions (e.g., narrow stacks or small diameter cylinders) tend to yield higher errors. The magnitude of these errors can be influenced by specific sensor characteristics, such as a wider FoV potentially being more susceptible to averaging errors over complex small shapes or a particular sensor’s internal signal processing being more or less robust to specular interference.

**Figure 14:**
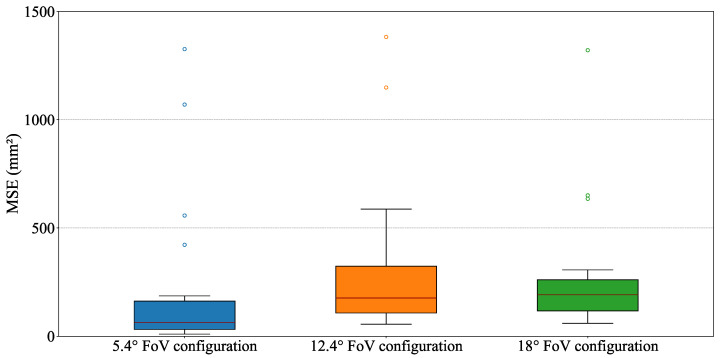
Total mean square error distribution of all combined measurements for each of the three sensors configurations.

An aggregate perspective on the consistency and magnitude of geometric perception errors across the different dToF sensor configurations we examined is provided by the Mean Squared Error (MSE) distributions presented in Figure 14. The dToF sensor configuration utilizing the narrowest FoV (5.4°) generally exhibited a lower median MSE and a more compact error distribution. This suggests that a narrower FoV tends on the whole to contribute to more consistent and lower average errors in geometric perception for the tested conditions. The two configurations employing wider FoVs (12.4° and 18°) showed broadly comparable median MSEs, which were generally higher than that of the narrowest FoV configuration; however, the 12.4° configuration displayed a notably wider spread in its MSE values, indicating greater variability in its measurement accuracy across the diverse range of tested scenarios. Overall, these aggregated results reinforce the general observation that dToF sensors designed with or operating in a mode that provides a narrower field of view tend to demonstrate more stable and on average more accurate geometric perception across the types of objects and environmental setups examined in this study, provided that other challenging factors such as extreme surface reflectivity are adequately managed by the sensor’s design and processing.

## 5. Conclusions

The primary aim of this research was to deepen the understanding of how direct time-of-flight sensors perceive their surroundings, with a specific examination of their interpretation of geometric forms, the influence of surface material properties such as reflectivity, and the impact of spatial arrangements. Through systematic experiments utilizing dToF sensors with varied optical characteristics and operational parameters, this work identified fundamental factors inherent to dToF sensing technology that significantly influence the reliability and precision of environmental perception.

Key perceptual dependencies were identified, revealing that a sensor’s field of view critically governs geometric accuracy. While narrower FoVs were observed to yield finer detail in shape recognition and better gap resolution, wider FoVs inherently introduce spatial averaging that, although smoothing features, sometimes increased stability against certain specular reflection artifacts. Critically, surface reflectivity, especially from specular materials, was found to introduce significant perceptual distortions, the manifestation of which can be modulated by the sensor’s FoV and internal signal processing. Observed variations in dToF perceptual capabilities extend beyond optical parameters such as FoV, stemming also from underlying sensor architectures and internal signal processing, which collectively dictate how environmental data is captured and interpreted.

These factors create inherent tradeoffs in dToF perception: enhancing geometric resolution can increase sensitivity to surface artifacts such as specular reflections, while achieving stability against such artifacts may reduce perceptual detail. Therefore, a clear understanding of these dToF perceptual characteristics is crucial for selecting and configuring sensors according to specific application requirements and environmental challenges, particularly in robotic safety. The comprehensive evaluation presented here can aid in designing safer and more reliable robotic systems through informed sensor selection and integration strategies.

Looking forward, our future work involves implementing and validating a practical robotic safety system. This system will be built upon the insights from this study, leveraging a combined sensing strategy that capitalizes on the distinct characteristics of different FoV configurations. The objective is to trigger appropriate robot behaviors in human–robot collaboration scenarios, thereby demonstrating a direct application of this study’s insights.

## Figures and Tables

**Table 1:** Overview of the measured objects used in the experiments. For cuboids, the dimensions are width (W), depth (D) and length (L); for cylinders, diameter (D) and height (H); for the ramp, width (W), height (H) and length (L).

Name	Shape	Material	Dimensions (mm)
Cuboid	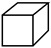	uncoated wood coated wood plastic aluminum	W: 75, D: 75, L: 150 W: 75, D: 75, L: 150 W: 75, D: 75, L: 150 W: 45, D: 45, L: 150 W: 75, D: 75, L: 150
Cylinder	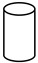	plastic	D: 100, H: 150 D: 75, H: 150 D: 50, H: 150 D: 35, H: 150 D: 25, H: 150
Ramp	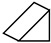	plastic	W: 100, H: 45, L: 150

**Table 2:** Scenarios ranked by overall performance for each sensor configuration, from best (top) to worst (bottom).

5.4° FoV Configuration	12.4° FoV Configuration	18° FoV Configuration
Cylinder 35	Hor 4	Hor 5
Hor 5	Hor 5	Hor 4
Hor 4	Hor 3	Gap 0
Gap 0	Cylinder 25	Hor 3
Hor 3	Multiple Shapes	Cylinder 25
⋮	⋮	⋮
Vert 3	Coated Wood	Plastic
Plastic	Gap 20	Vert 2
Vert 4	Vert 3	Aluminum
Coated Wood	Vert 4	Vert 3
Aluminum	Aluminum	Vert 4

## Data Availability

Data are contained within the article.

## References

[1] Amad-ud-Din., Halin I.A., Shafie S.B. A review on Solid State time of flight TOF range image sensors. Proceedings of the 2009 IEEE Student Conference on Research and Development (SCOReD).

[2] Bamji C., Godbaz J., Oh M., Mehta S., Payne A., Ortiz S., Nagaraja S., Perry T., Thompson B. (2022). A Review of Indirect Time-of-Flight Technologies. IEEE Trans. Electron Devices.

[3] Gyongy I., Dutton N.A.W., Henderson R.K. (2021). Direct Time-of-Flight Single-Photon Imaging. IEEE Trans. Electron Devices.

[4] Scholz C., Cao H.L., Imrith E., Roshandel N., Firouzipouyaei H., Burkiewicz A., Amighi M., Menet S., Sisavath D.W., Paolillo A. (2024). Sensor-Enabled Safety Systems for Human–Robot Collaboration: A Review. IEEE Sens. J..

[5] Wunderle Y., Lyczkowski E. Sensor fusion for functional safety of autonomous mobile robots in urban and industrial environments. Proceedings of the 2022 IEEE 27th International Conference on Emerging Technologies and Factory Automation (ETFA).

[6] (2024). Intel Corporation, Santa Clara, CA. Intel^®^ RealSense™ D400 Series Product Family Datasheet. Document Number: 335167-012, Rev. 012. https://cdrdv2-public.intel.com/841984/Intel-RealSense-D400-Series-Datasheet.pdf.

[7] Kumar S., Arora S., Sahin F. Speed and Separation Monitoring using On-Robot Time-of-Flight Laser-ranging Sensor Arrays. Proceedings of the 2019 IEEE 15th International Conference on Automation Science and Engineering (CASE).

[8] Liang J., Wu J., Huang H., Xu W., Li B., Xi F. (2019). Soft Sensitive Skin for Safety Control of a Nursing Robot Using Proximity and Tactile Sensors. IEEE Sens. J..

[9] Yim H., Kang H., Nguyen T.D., Choi H.R. (2024). Electromagnetic Field & ToF Sensor Fusion for Advanced Perceptual Capability of Robots. IEEE Robot. Autom. Lett..

[10] SICK AG (2024). End-of-Arm-Safeguard—Collision Protection Around the Gripper for Safe Human-Robot Collaboration. https://www.sick.com/sg/en/catalog/products/safety/safety-systems/end-of-arm-safeguard/c/g585667.

[11] Laurenzis M., Marić A., Bacher E., Pietrzak M., Schertzer S., Grella F., Calinon S., Secchi C., Marconi L. (2024). Sparse Optical Sampling in the Close Proximity of a Robotic Arm. Proceedings of the European Robotics Forum 2024.

[12] Wang C., Han Q., Li J., Li C., Zou X. (2024). YOLO-BLBE: A Novel Model for Identifying Blueberry Fruits with Different Maturities Using the I-MSRCR Method. Agronomy.

[13] Adamides O.A., Modur A.S., Kumar S., Sahin F. A Time-of-Flight On-Robot Proximity Sensing System to Achieve Human Detection for Collaborative Robots. Proceedings of the 2019 IEEE 15th International Conference on Automation Science and Engineering (CASE).

[14] Himmelsbach U.B., Wendt T.M., Hangst N., Gawron P. Single Pixel Time-of-Flight Sensors for Object Detection and Self-Detection in Three-Sectional Single-Arm Robot Manipulators. Proceedings of the 2019 Third IEEE International Conference on Robotic Computing (IRC).

[15] Yoshida T., Golyanik V., Wasenmuller O., Stricker D. Improving Time-of-Flight Sensor for Specular Surfaces with Shape from Polarization. Proceedings of the 2018 25th IEEE International Conference on Image Processing (ICIP).

[16] Zámečníková M., Wieser A., Woschitz H., Ressl C. (2014). Influence of surface reflectivity on reflectorless electronic distance measurement and terrestrial laser scanning. J. Appl. Geod..

[17] Ronchini Ximenes A., Padmanabhan P., Lee M.J., Yamashita Y., Yaung D.N., Charbon E. (2019). A Modular, Direct Time-of-Flight Depth Sensor in 45/65-nm 3-D-Stacked CMOS Technology. IEEE J.-Solid-State Circuits.

[18] Gutierrez-Barragan F., Chen H., Gupta M., Velten A., Gu J. (2021). iToF2dToF: A Robust and Flexible Representation for Data-Driven Time-of-Flight Imaging. IEEE Trans. Comput. Imaging.

[19] Baek E.T., Yang H.J., Kim S.H., Lee G., Jeong H. (2020). Distance Error Correction in Time-of-Flight Cameras Using Asynchronous Integration Time. Sensors.

[20] STMicroelectronics (2024). VL53L4CD Time-of-Flight High Accuracy Low Power Proximity Sensor—Datasheet.

[21] Broadcom Inc. (2022). AFBR-S50MV85I Time-of-Flight Sensor Module for Distance and Motion Measurement—Datasheet.

[22] Botchkarev A. (2018). A new typology design of performance metrics to measure errors in machine learning regression algorithms. Interdiscip. J. Inf. Knowl. Manag..

[23] Lambers M., Kolb A. (2018). Quantified, Interactive Simulation of AMCW ToF Camera Including Multipath Interference and Noise. Sensors.

[24] Yang C., Kang J., Eom D.S. (2024). Enhancing ToF Sensor Precision Using 3D Models and Simulation for Vision Inspection in Industrial Mobile Robots. Appl. Sci..

[25] Zhao Y., Zhang X., Li H. (2023). Deep Learning for Generating Time-of-Flight Camera Artifacts. Sensors.

[26] He Y., Liang B., Zou Y., He J., Yang J. (2017). Depth Errors Analysis and Correction for Time-of-Flight (ToF) Cameras. Sensors.

